# Ultrasound-Guided Percutaneous Renal Biopsy in 295 Children and Adolescents: Role of Ultrasound and Analysis of Complications

**DOI:** 10.1371/journal.pone.0114737

**Published:** 2014-12-09

**Authors:** Mareike Franke, Annette Kramarczyk, Christina Taylan, David Maintz, Bernd Hoppe, Friederike Koerber

**Affiliations:** 1 Department of Radiology, University Hospital Cologne, Cologne, Germany; 2 Department of Pediatrics, University Hospital Cologne, Cologne, Germany; 3 Department of Pediatrics, University Hospital Bonn, Bonn, Germany; University Hospital Oldenburg, Germany

## Abstract

**Introduction:**

Percutaneous renal biopsy (PRB) is a decisive diagnostic procedure for children and adolescents with renal diseases. Aim of this study was to evaluate retrospectively the complication rates of percutaneous kidney biopsies and their therapeutic consequences to assess the role of ultrasound-guidance including Doppler ultrasound examinations in preparation, execution and follow-up care and to present a recommended protocol.

**Patients and Methods:**

Institutional review board approved this retrospective study; informed consent was waived. Between 1997 and 2011 a total of 438 ultrasound-guided biopsies were performed in 295 patients, 169 of the biopsies were performed on kidney transplants. Average age of patients was 10.2+/−5.2 years (range of 15 days until age of 23). Before and post biopsy ultrasound examination including Doppler examination was carried out. Biopsy itself was ultrasound monitored. Complications were analysed with regard to age of patient, kidney transplants, year of occurrence, number of punctures, performing physician and time interval of occurrence to develop an optimized protocol for ultrasound-guidance.

**Results:**

In 99% of cases successful PRB were performed, i.e. enough kidney parenchyma for histological analysis was obtained. No lethal or major complication that required surgical intervention occurred. Eighteen relevant complications were observed (complication rate: 4.1%). Except in one case in which additional MRI diagnostic was necessary, ultrasound examination after 4 hours post biopsy or even earlier when symptoms occurred, was able to detect complications and determine indications for intervention.

**Conclusion:**

Ultrasound-guided PRB is an established and effective method in children and adolescents, but shows a certain rate of complications and therefore should not be indicated without diligence. Ultrasound including Doppler ultrasound is a valuable tool in preparation, guidance of biopsy, detection of complications and in follow-up care. Ultrasound examinations (including Doppler) pre-, during and 4 hours post kidney biopsy and, depending from case, a few days until weeks after biopsy is recommended.

## Introduction

Percutaneous renal biopsy (PRB) is an established diagnostic method and has been performed for plenty of years [1]. Even in children and adolescents percutaneous renal biopsy is a routine procedure in paediatric nephrology. Complications of percutaneous kidney biopsies range from small perirenal hematomas to bleedings that require blood transfusions and even to loss of kidney. In very rare cases death of patients was described [2].

Ultrasound-guidance of renal biopsy is an often used but not yet generally regarded standard procedure. Data shown in a multi-centre study performed on behalf of the British Association of Paediatric Nephrology emphasize the need of protocol standardization for renal biopsies that allows comparing success and complications [3]. However, too little has been achieved so far to design a standard for ultrasound-guidance for renal biopsies.

The purpose of our study was to analyse complications with regard to age of patient, kidney transplants, year of occurrence, number of punctures, performing physician and time interval of occurrence post biopsy to develop an optimized protocol for ultrasound-guidance in preparation, execution and follow-up care of biopsies.

## Patients and Methods

### Patients

This retrospective study was approved by the ethics committee of the Medical Faculty of the University of Cologne, which waived the requirement to prove informed consent. Since the study is purely retrospective, no further ethics considerations apply.

In the Department of Paediatrics of the University Hospital of Cologne kidney biopsies have been performed ultrasound-guided since 1993. From 1997 until 2005 biopsies were taken by two trained pediatric nephrologists, since 2005 residents in training were involved.

**Figure 1:**
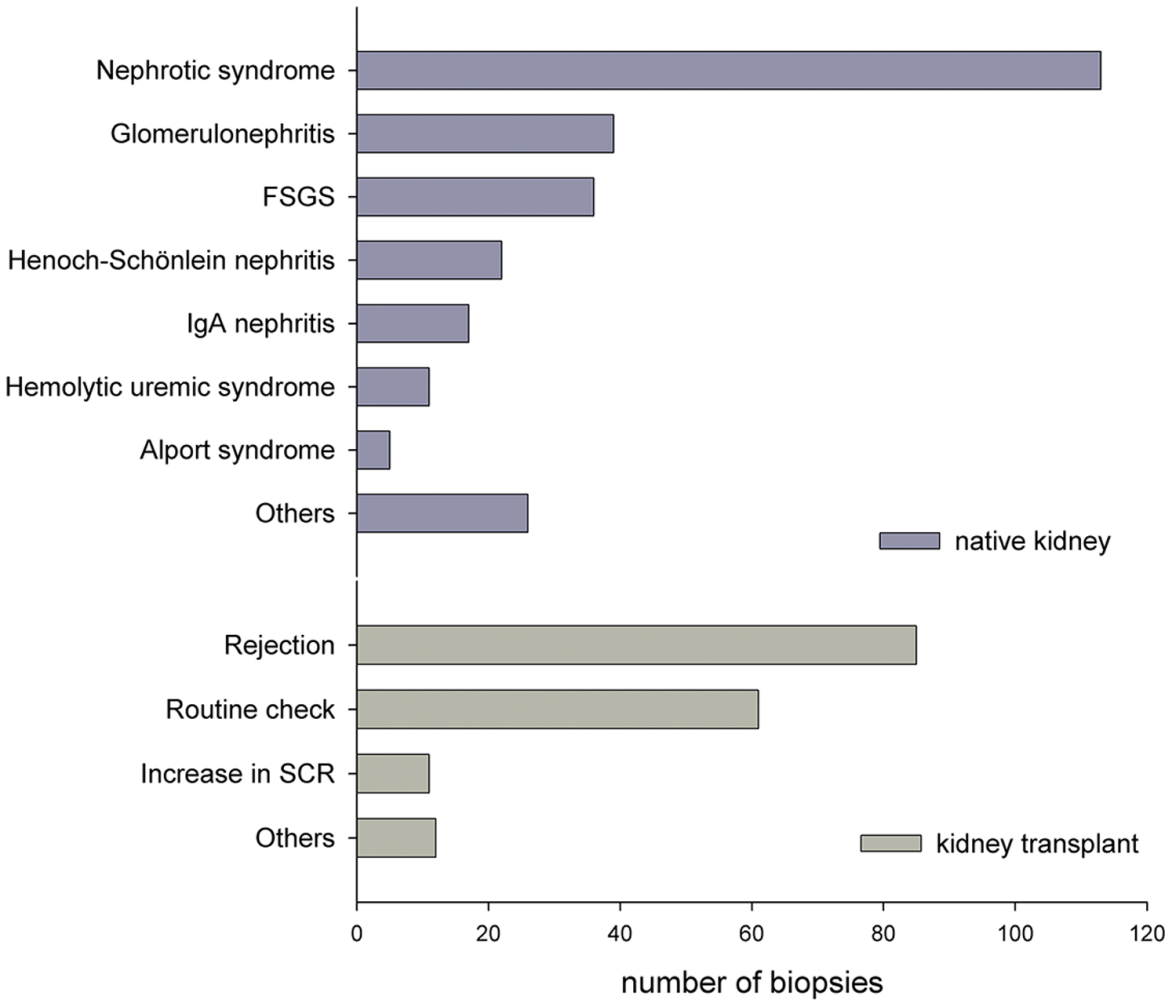
Indications for biopsies in 295 children and adolescents −169 of the biopsies were performed on kidney transplants (FSGS – focal and segmental glomerulosclerosis, SCR – serum creatinine).

Between 1997 and 2011 a total of 438 ultrasound-guided biopsies were performed in 295 patients (164 boys and 131 girls), 169 of the biopsies were performed on kidney transplants. The average age of patients was 10.2+/−5.2 years (range of 15 days until age of 23). Indications for kidney biopsies are shown in Fig. 1.

### Methods

In preparation of kidney biopsy ultrasound and Doppler ultrasound examination of kidneys were performed in each patient, independent of native kidneys or kidney transplants. In these examinations kidneys, the retro-peritoneum and the bladder were inspected to assess possible biopsy difficulties and anatomical orientation.

Depending from native kidney or kidney transplant, patients were in prone or in dorsal position during biopsy. Biopsies were carried out ultrasound-guided in local anaesthesia using “Core Tissue Biopsy Needles” (14–16 Gauge). Depending from age and compliance patients got a short anesthesia.

Until 2007 ultrasound guidance of biopsies was performed with an *Acuson 128 XP* ultrasonic device, since 2007 a *GE Logic 7* ultrasonic device was used. Examinations pre- and post-biopsy were carried out until 2003 with an ultrasonic device type *Acuson 128 XP* (Acuson, Mountain View, CA) or since 2003 with an ultrasonic device type *Sequoia* (Siemens, Erlangen, Germany), since 2007 additionally the *GE Logic 7* ultrasonic device was used. Vector or sector transducers (3.5 to 5 MHz) or for high resolution linear array transducers with higher frequencies (7.5 to 10 MHz) were used.

As well as for pre-biopsy, post-biopsy examination of both kidneys with volumetric analysis and B-mode ultrasound was performed; also an examination of bladder and an examination of the retroperitoneal space were accomplished. Since first vascular complications were observed in 2003 colour Doppler examinations were performed mandatorily.

#### Ultrasound-Guidance of Biopsies

**Figure 2:**
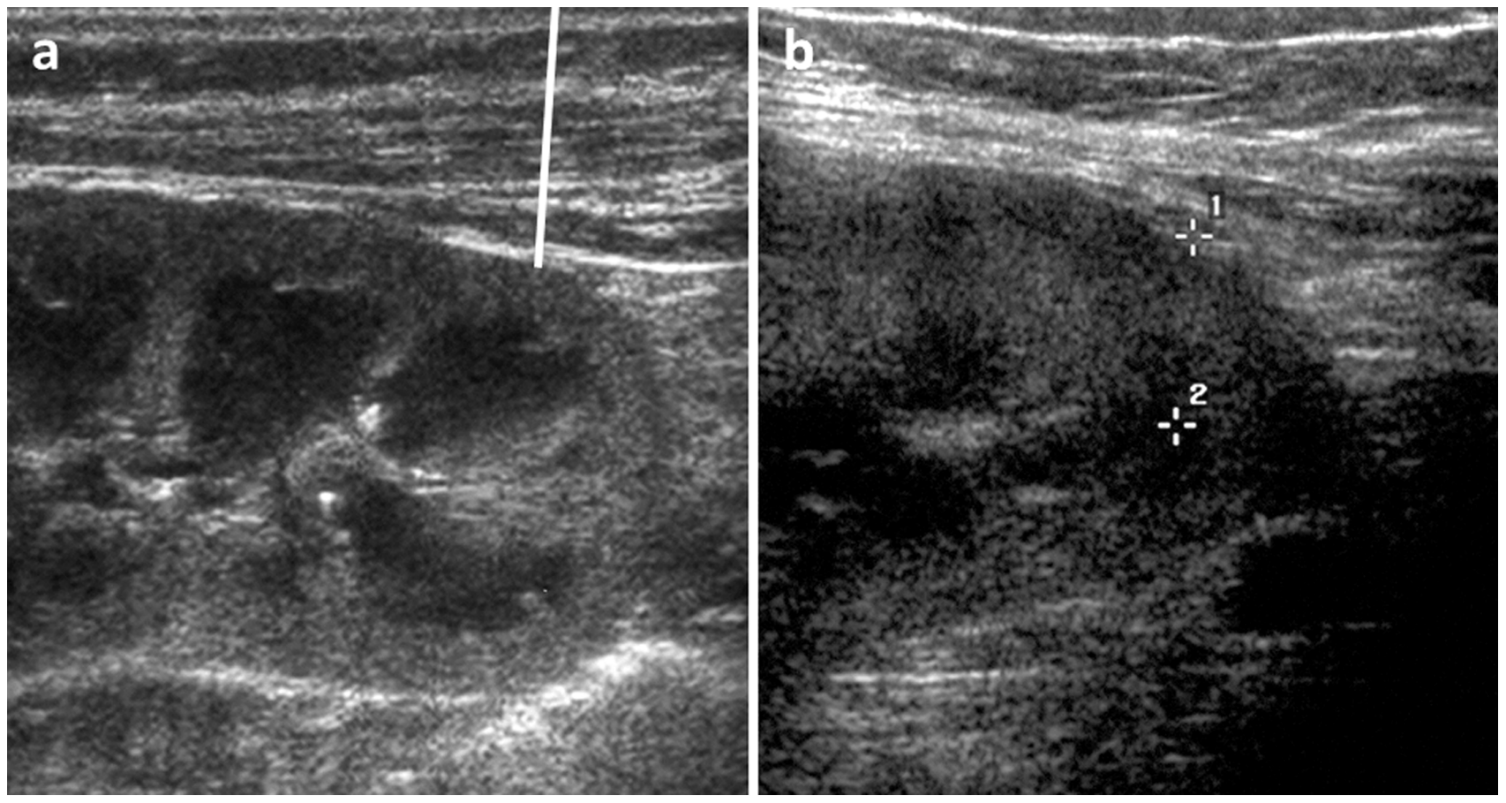
B mode sonographic image of a kidney. The distance between the lower kidney pole, the destined localization of biopsy and the skin was determined. Depth of biopsy was aligned on the measured skin-kidney pole distance (white line).

**Figure 3:**
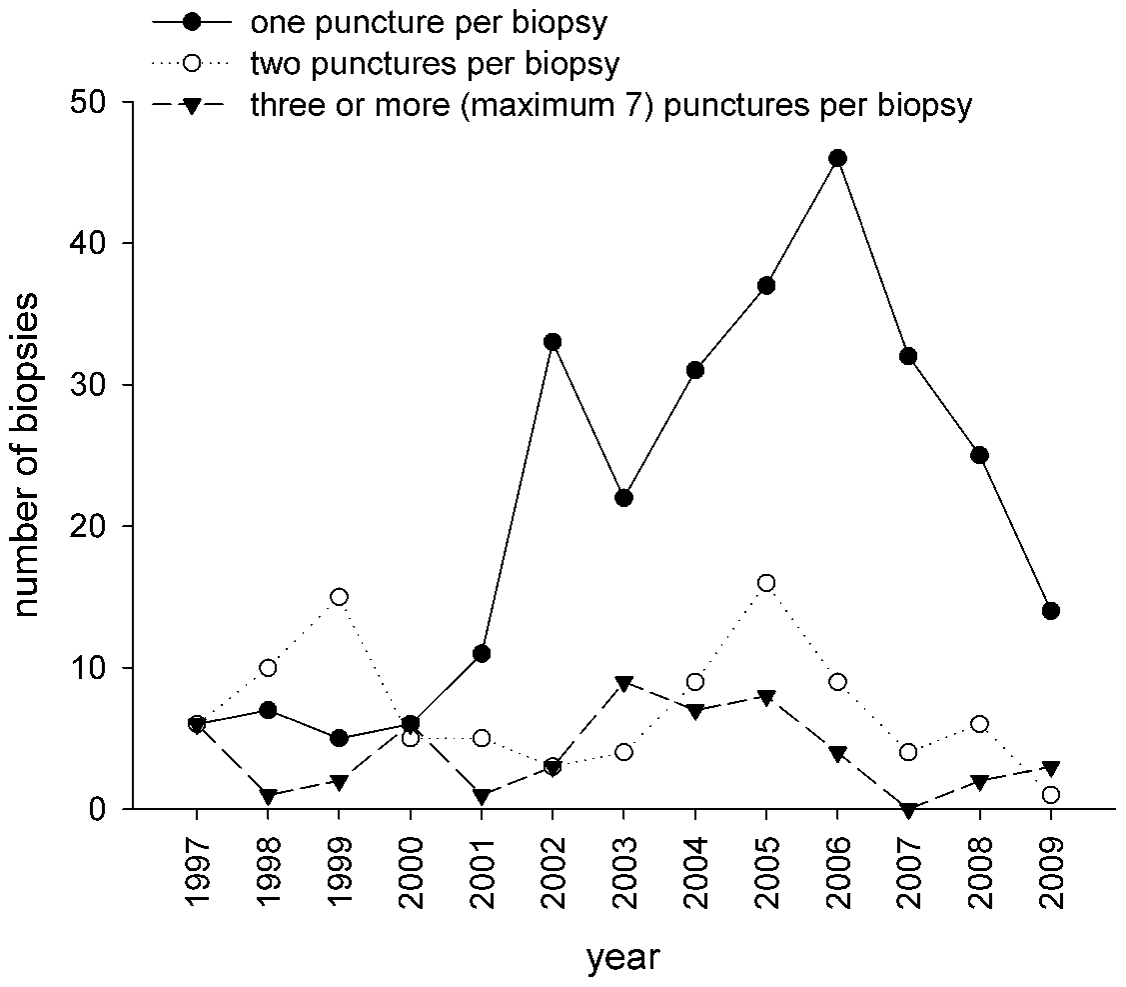
Number of punctures per biopsy.

Pre-biopsy and during biopsy the distance between the lower kidney pole, the destined localization of biopsy and the skin was determined (see Fig. 2). In rare cases, when the distance was too long, it was necessary to use a sector or convex transducer. Afterwards the puncture site was labelled on the skin. To avoid displacement of kidney (especially native kidneys show a breath-depending displacement) labelling was carried out with in breath-holding. After disinfection, sterile covering, anaesthesia of the skin and a small puncture incision, puncture was performed with the ultrasound transducer (in sterile cover) in parallel with the puncture direction. The needle was continued to advance until it touches the lower pole of the kidney, depth of biopsy was aligned on the measured skin-kidney pole distance (see Fig. 2). Afterwards kidney was punctured and the containing material was determined. Number of needed punctures during kidney biopsies were registered until 2009 (see Fig. 3).

#### Follow-up Examinations

After a bandage was applied a sandbag was put on the puncture site until first ultrasound examination. From 1997 until 2003 bed rest for 24 hours was necessary and ultrasound examination after 4 and 24 hours was done.

From 2003 until 2011 when biopsies were performed on an outpatient’s basis, bed rest for only 4 hours was performed; ultrasonic examination was done 4 hours after biopsy, if negative patients were able to go home. If positive e.g. for bleeding or AV fistula, or if laboratory values were abnormal, e.g. declining haemoglobin levels, patients were admitted for further controls.

#### Data Analysis

Slim, often falciform, perirenal hematomas until a width of approx. 2.5 cm, hematomas along the puncture channel and moderate pain were considered as normal consequences of the biopsy and were not regarded as complications. “Relevant complications“ were:

- Perirenal or retroperitoneal bleedings causing a drop in hemoglobin of more than 10% or hematomas with a size of more than 2.5 cm width
- Bleedings into the renal pelvis and urinary bladder
- Arteriovenous fistulas in the puncture area (with or without bleedings)

All data were collected and analysed retrospectively. Complications were analysed with regard to therapeutic consequences, age of patient, kidney transplants, year of occurrence, number of punctures, performing physician and time interval of occurrence. The focus of this analysis was the role of ultrasound examinations in pre-biopsy examination, ultrasound-guidance of biopsy and follow-up examinations.

#### Statistics

Association of number of complication and time period (either 1997–2002 vs 2003–2011 or 1997–2004 vs 2005–2011) or number of punctures per biopsy and time period were evaluated by Pearson’s chi-square test or Fisher’s exact test, contingent on expected cell counts below 5. Moreover, the association with the number of complications was adjusted for age, gender and kidney transplant by multiple logistic regression. We could not adjust for 30 different indications, nor could we identify a sensible subgrouping. The strength of association is summarized by the odds ratio. Calculations were done with SPSS Statistics (IBM Corp., Armonk, NY, USA).

## Results

In 99% of cases biopsies were performed successfully, i.e. enough kidney parenchyma for histological analysis was obtained.

### Complications

No lethal complication occurred. No permanent loss or decrease of renal function was observed. Four specific groups of complications were distinguished (table 1):

**Table 1:** Eighteen relevant complications occurred among 438 biopsies (complication rate: 4.1%), with 6 patients needing an angiographic or cystoscopic intervention (1.3%), 3 patients receiving a blood transfusion (0.6%) and complications showed spontaneous recovery in 9 patients (2%).

	Total number	Kidney transplants	Native kidneys	Therapeutic consequences	Spontaneous recovery
**Perirenal/retroperitoneal bleeding**	**6**	2	4	3 x blood transfusion	3
**Bleeding in the urinary tract**	**5**	1	4	1 x cystoscopy	4
**AV fistula without bleeding**	**5**	5	0	3 x angiographic intervention	2
**AV fistula with bleeding in the urinary tract**	**2**	1	1	2 x cystoscopy plus angiographic intervention	0
**total**	**18**	**9**	**9**	**3 blood transfusions, 6 interventions**	**9**

**Figure 4:**
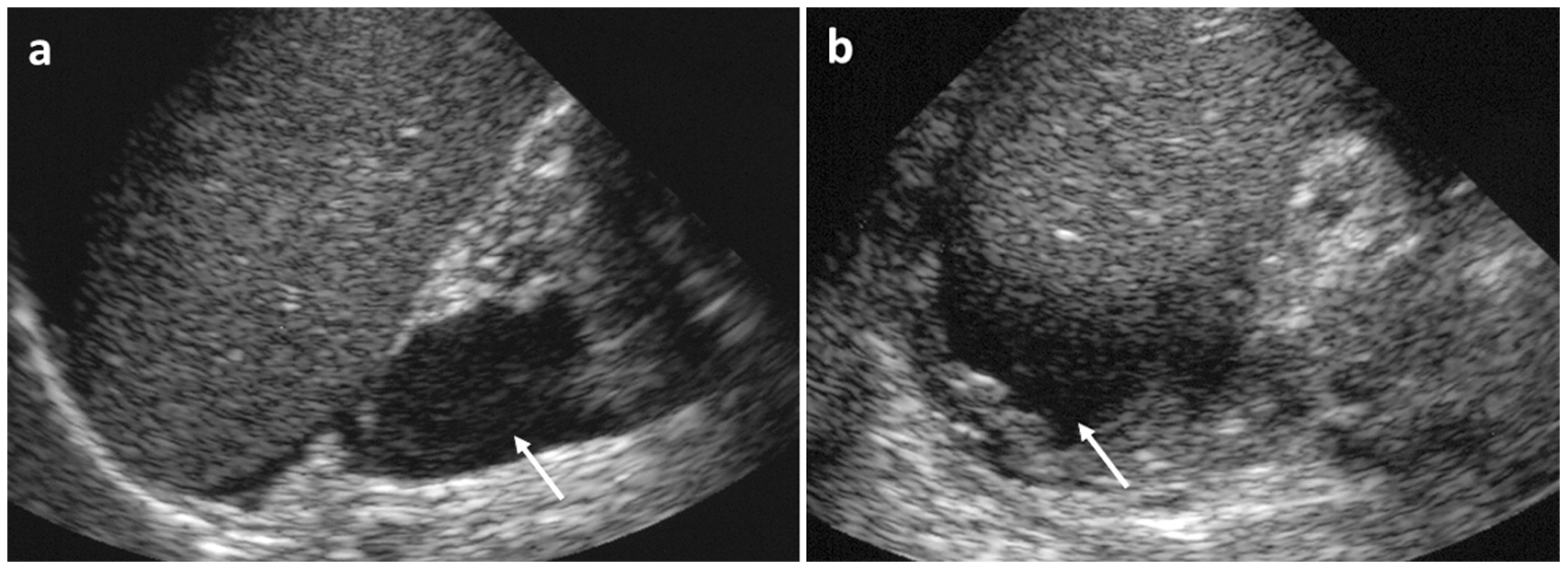
Abdominal bleeding in a six year old male patient 4 hours after biopsy, which caused a drop of haemoglobin. White arrow indicates perirenal hematoma.

**Figure 5:**
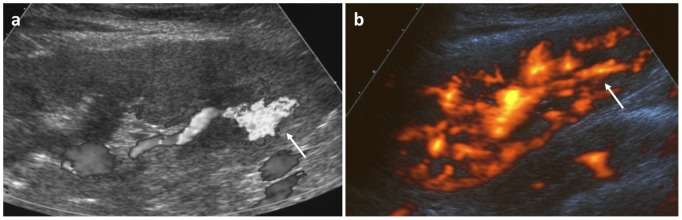
Ultrasound findings of an AV fistula (white arrow). The fistula showed spontaneous regression (13 year old patient, female).

**Figure 6:**
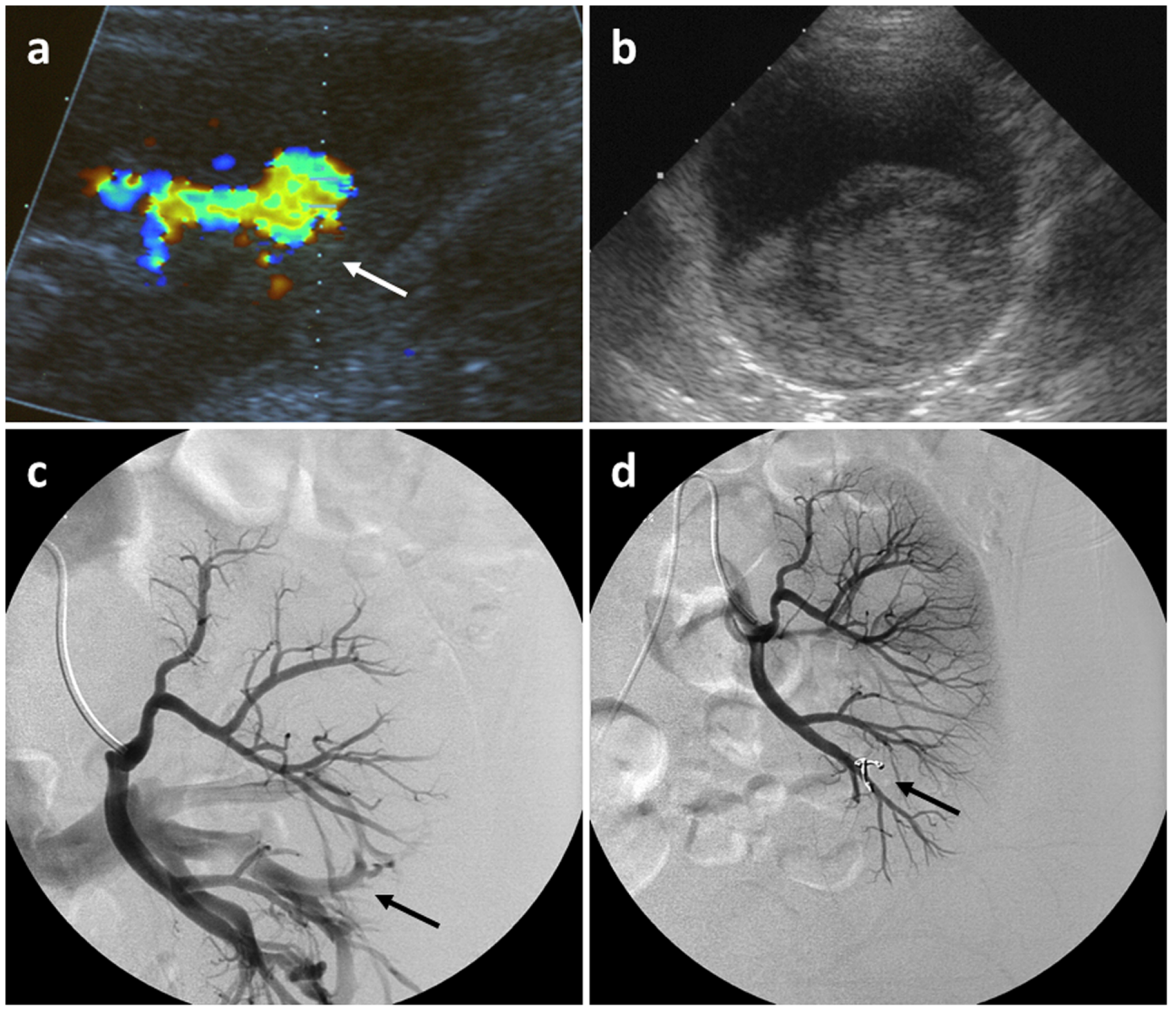
Ultrasound findings of an arteriovenous fistula (a, white arrow) and a bladder tamponade (b). AV-fistula was angiographically embolized (c and d, black arrow) (10 year old patient, male).

1. *Perirenal/retroperitoneal bleeding with a drop in hemoglobin of more than 10% (n = 6;* *Fig. 4* *).*
  Three patients received a blood transfusion but none of the patients needed interventional treatment. One patient showed clinical symptoms of an acute abdomen and CRP values increased rapidly. Clinical improvement after an antibiotic treatment was observed. Except for one patient, in whom bleeding was observed during first night after biopsy, bleeding occurred within the first four hours. The hematomas were detected sonographically and their size monitored sonographically.
2. *Bleeding in the urinary tract with hematuria or blood clots in the urinary bladder (n = 5).*
  With one exception in which a patient had a drop of hemoglobin after three weeks (which was possibly not related to kidney biopsy), patients with this complication were clinically apparent within four hours. In one case it was necessary to cystoscopically remove a bladder tamponade, the other cases showed spontaneous recovery. The size of blood clots and bleeding was monitored sonographically. Ultrasound examination methods were employed to monitor/detect urinary obstructions.
3. *Arterio-venous (AV) fistula without relevant bleeding (n = 5,* *Fig. 5* *).*
  Except from one case, fistulas were detected sonographically in the follow-up examination after four hours. In one patient without acute symptoms the AV fistula was discovered in a sonographic control examination after three months. Because of sonographic findings and a slightly decreased general condition and increase in serum creatinine, an interventional embolization was performed. In two patients fistulas showed a spontaneous recovery within a few months. One AV-fistula occurred in a kidney transplant with chronic transplant nephropathy; because of sonographic findings and increase of serum creatinine arterial embolization was performed.
  In the last case with AV-fistula arterial embolization was promptly necessary based on distinct sonographic findings: AV fistula at the lower pole of the left kidney with a large draining vein and an arterial signal (resistance index between 0.5 until 0.57).

1. *AV-fistulas with relevant bleedings in the urinary tract (n = 2,* *Fig. 6* *).*
2. In these two cases patients showed aggravated pain and hematuria within two hours after biopsy. The AV-fistula was detected by ultrasound; in addition a bladder tamponade was found, which threatened to cause an intrarenal obstruction. Cystoscopical removal of the bladder tamponade and angiographic embolization of the AV-fistula was performed in both patients.

In total eighteen relevant complications occurred, adding to a complication rate of 4.1% (see table 2). Eleven bleeding episodes were observed (2.5%), six perirenal/retroperitoneal bleedings (1.3%), five bleedings in the lower urinary tract (1.1%). Seven AV-fistulas occurred (1.5%), two of them with bleeding in the urinary tract (0.4%).

**Table 2:** Eighteen relevant complications occurred among 438 biopsies (complication rate: 4.1%).

	AV fistula w/ointerv. treatment	AV fistula withinterv. treatment	Bleeding w/ointerv. treatment	Bleeding withinterv. treatment	total
**Native kidney**	0	1	7	1	**9**
**Kidney transplant**	2	4	3	0	**9**
**total**	**2**	**5**	**10**	**1**	**18**

Six patients needed an angiographic or cystoscopic intervention (1.3%).

Six post biopsy complications (five AV-fistulas and one bleeding) needed an angiographic or cystoscopic intervention (1.3%). Two AV-fistulas and ten bleedings showed a spontaneous regression (see table 2). Six of the seven AV-fistulas appeared in kidney transplants.

**Figure 7:**
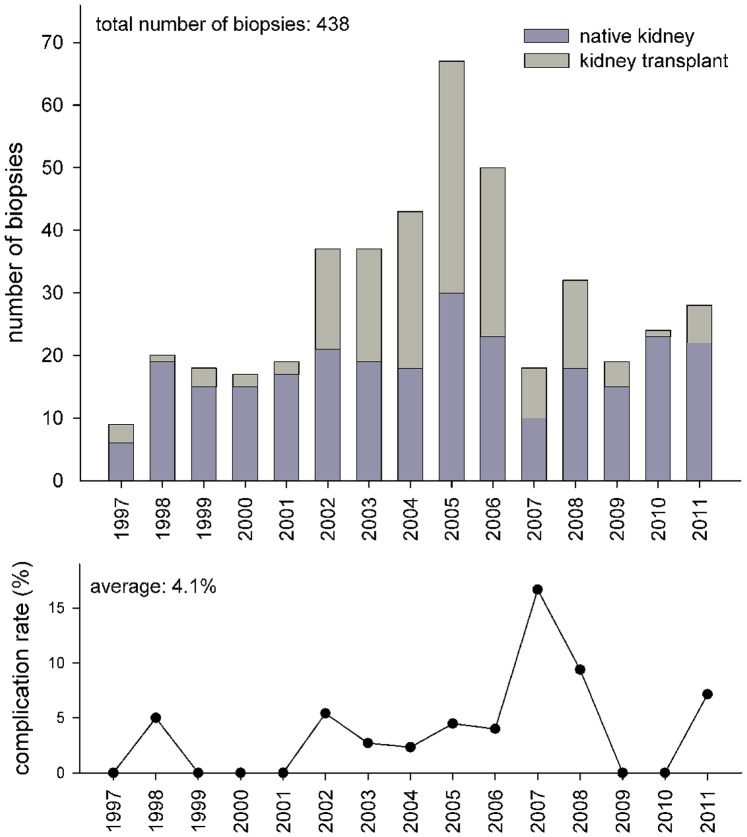
Between 1997 and 2011 a total of 438 ultrasound-guided biopsies were performed in 295 patients.

Time course of annual number of biopsies and annual complication rate is shown in Fig. 7. Three complications each appeared in 2005, 2007 and 2008, with the highest complication rate in 2007 (16.6%) and 2008 (9.4%). From 1997 until 2006 the complication rate was low with zero to 5.4%.

From 1997 until 2002 when biopsies were performed with hospitalization the overall complication rate was 2.5%, from 2003 until 2011, when biopsies were performed on an outpatient’s basis, complication rate showed a little increase up to 4.7%, which was not statistically significant (p = 0.421, odds ratio 1.9 with 95% confidence interval 0.55 to 6.8). However, we arguably observed a clearer trend towards a higher complication rate in 2005–2011 (5.5%) compared with 1997–2004 (2.5%), when residents in training were involved in kidney biopsies (p = 0.120, odds ratio 2.3 with 95% confidence interval 0.79 to 6.4). When adjusting for possibly confounding variables (i.e. age, gender, kidney transplant) this “trend” did not change (2.2, 0.78 to 6.5), but remained below statistical significance. Moreover, we could not find an association of incident complication and number of punctures (p = 0.938, exact Wilcoxon rank sum test). In fact, in the 13 biopsies with 4 to 7 punctures no complication occurred.

### Assessment

For one case of an AV fistula which was very complex and indication for interventional treatment was not clear, additional MRI imaging was carried out. Except from this case angiography or cystoscopy were indicated because of ultrasound findings without further imaging. Most complications were found in ultrasound examination after 4 hours post biopsy or even earlier, when symptoms occurred.

## Discussion

Percutaneous ultrasound-guided renal biopsy is an established and effective diagnostic method in children and adolescents since many years [4], [5].

Despite reduction of complication rates and improvement of safety based on the use of ultrasound guidance [6], kidney biopsies still have a certain rate of complications and therefore should not be done without diligence. Complications range from small perirenal hematomas, bleedings that cause obstruction of the urinary tract to AV fistulas with persistent macrohematuria and obstructive blood clots [7], [8]. According to literature and based on different definitions the complication rate fluctuates from 0 to 45% [5], [9], [10], [11].

Standards of the British Association of Pediatric Nephrology (BAPN) indicate that the complication rate should not be above 5% [12]. However, data of a recent multicenter study [3] showed that the average complication rate is approximately 10.4% and therefore much too high. This emphasizes the need of a standard operation procedure, both for indication of biopsy and staff training in kidney biopsy procedures. An important aspect is that in some of the pre-existing studies information is in part based on clinical symptoms and not on imaging procedures like ultrasound [13], [4], [14]. The ultrasound guidance of kidney biopsies was used in most studies for biopsy monitoring but the sonographic post biopsy assessment (including Doppler examinations) is obviously not always part of a standard operation procedure.

In our patients we observed a complication rate of 4.1% and thereby fulfilled the standards of BAPN. We monitored eleven bleedings (2.5%) and seven AV fistulas (1.5%). An interventional treatment was necessary in six patients (one bleeding and five AV fistulas). Spontaneous recovery was observed in two patients with an AV fistula. We performed an ultrasound guidance of biopsies and we had a prospective and strict protocol for ultrasound follow-up examinations, including Doppler ultrasound.

The number of biopsies increased from 1997 to 2005 (see Fig. 7). During this time period especially the number of kidney transplant biopsies increased, whereas the number of biopsies of native kidneys remained almost constant. Decreased number of biopsies from 2007 until 2011 is due to a more restricted discussion of biopsy indications, based on the complications seen in 2007 and 2008. The number of needed punctures is shown in Fig. 3. Even during 2007 and 2008, when the highest complication rate was observed, most biopsies were performed with one puncture. So we were able to show that there was no relevant correlation between number of punctures during biopsies and complications.

Provided that the biopsy was performed with a functioning team of physicians there was no correlation between the person who performed the biopsy (e.g. pediatric nephrologist or resident) and the complication rate. The only “trend” observed was the frequent changes within the biopsy team since 2005, which could explain the increase in complication rate in 2007–2008. However, the complications could have also occurred as a coincidence because of the patient collective in these years, e.g. two patients with relevant bleedings had an increased bleeding disposition. Changes in the biopsy team also occurred in the further years, but from 2008 until 2010 no relevant complications that needed interventional treatment were observed. A statistical significant difference in complication rate was not observed comparing inpatient’s and outpatient’s biopsies. The total number of successful biopsies in our study corresponds to the standards of the BAPN. These standards indicate that in at least 95% of biopsies enough kidney parenchyma must be collected for histological analysis and that there should not be a difference between different punctures during biopsy.

One of the largest studies about renal biopsies depicted data of 9288 biopsies (715 children and 8573 adults) [15]. In this large study, collecting data from 1988–2010, no biopsies of kidney transplants were included and a complication rate of 2.1% was observed. However, the definition of complications used in that study only included gross hematuria, the need of blood transfusion, of surgery or arterial embolization. In this large study the number of (large) perirenal hematomas was not part of the total complication rate. Furthermore only in one case with an AV fistula the imaging technique was reported.

Reports about kidney biopsies in children and adolescents and their complication rates are hard to find and they consist of smaller patient collectives, compared to adult studies (see table 3). For example Sinha and colleagues [4] described their experiences with 186 biopsies (102 native kidneys, 84 transplant kidneys). A complication rate of 12% was observed. Most of the complications were macrohematuria (7%). No ultrasound follow-up examination was done or reported.

**Table 3:** Synopsis of literature.

	Number ofpatients	Age of patients		kidneys		Complications* (%)		Interventions(%)
		adults	children	native	KTP	Total*	AV fistula	
**Riccabona (1998) ** [19]	50		x	x	x	32.0	12.0	n/a
**Bilge (1999) ** [18]	896		x	x		16.5	0.3	n/a
**Schwarz (2005) ** [23]	1670	x		x		13.2	7.5	1.0
**Nammalwar (2006) ** [10]	250		x	x		45.0	n/a	n/a
**Sinha (2006) ** [4]	185		x	x	x	12.0	–	1.5
**Sweeney (2006) ** [14]	65		x		x	21.0	–	–
**Stratta (2007) ** [13]	1387	x		x		24.0	0.1	2.2
**Torrez Munoz (2010) ** [11]	623	x		x		17.0	–	0.8
**Skalova (2010) ** [16]	166		x	x		23.5	–	–
**Printza (2011) ** [17]	84		x	x		11.0	n/a	–
**Tondel (2012) ** [15]	9288	x	x	x		2.1	0.001	n/a
**Own data**	438		x	x	x	4.1	1.5	1.3

(n/a – data could not be retrieved; KTP – kidney transplant; interv. – interventional treatment; * note: different definitions of complications are used).

There are a number of studies of kidney biopsies in children [16], [17], in which ultrasound or Doppler ultrasound follow-up examinations were not routinely done or when being part of the protocol no Doppler examinations were performed [10].

Very few studies in children and adolescents reported on AV fistulas [18]. Many studies did not [4], [10], [14], [16], [17]. In these studies cited above, only Nammalwar and colleagues [10] performed ultrasound follow-up but without Doppler examination. Here it is to speculate, that underdiagnosis especially of the smaller fistulas was based on the missing ultrasound and duplex examinations.

An explicit recommendation for Doppler examinations in children and adolescents was given by Riccabona and colleagues [19]. They used not only Doppler ultrasound examinations to proof the existence of an AV fistula, but also Doppler spectrum analysis to detect a reduction of perfusion in comparison to preceding examinations. Hence, we may conclude, that the detection of complications depends on the quality of post-biopsy assessment. AV-fistulas or hematomas may evade detection if no ultrasound is done post-biopsy. In particular for detecting fistulas, Doppler ultrasound is necessary. While many fistulas may be entirely asymptomatic, resolve spontaneously and hence were not reported in most studies cited, they may in some cases cause symptom-free damages like microhematuria [20] or an increase of serum creatinine ([21], own data). Even after many years asymptomatic AV fistulas may decompensate and get symptomatic by causing bleedings [22], or by developing a hemodynamic impact. For those reasons, it is very important to detect AV fistulas early and to monitor them frequently. When no spontaneous regression is detected, arterial embolization should be carefully considered, even when the fistula is asymptomatic, in order to prevent late onset of complications.

In general AV fistulas are more frequently seen in kidney transplants. For example, Bilge and colleagues [18] described two AV fistulas in transplant biopsies, but only one AV fistula in native kidneys in a study with 896 children (866 native kidneys, 30 transplant kidneys). Thereby the risk of experiencing an AV fistula was approximately 6.3% for transplant kidneys but only 0.11% for native kidneys.

This increased prevalence of AV fistula after transplant kidney biopsies was confirmed in our series: in six of the seven patients with this complication a kidney graft biopsy was performed. In contrast, relevant bleedings were observed mostly in patients after native kidney biopsies. So in our opinion it is important especially for transplanted patients to perform a Doppler ultrasound examination before kidney biopsy to exclude the possibility of a preexisting AV fistula that was caused by earlier biopsies and post biopsy to detect fistulas that were caused by the current biopsy.

There is a need to find the most adequate time interval for follow-up examinations, especially considering the differences in our outpatient treatment. A 4, 6 or even 11 hour time interval in which most of the relevant complications occurred were reported [14], [19], [23], [24]. Stratta and colleagues [13] found macrohematuria to be evident during the first 6 hours, however all hematomas were evident after 24 hours. AV fistulas were apparent after an even longer time interval. Sinha and colleagues [4] observed that relevant bleedings in the urinary tract manifest during the first 4 until 6 days after biopsy, catheterization because of bladder tamponade was necessary until 48 hours post-biopsy.

But also late complications like AV fistula are possible, with AV fistulas appearing days until weeks after biopsy until very late occurrences up until 15 months post biopsy [25]. In our series one case of severe bleeding occurred three weeks after biopsy (drop of hemoglobin below 3 g/dl).

In a multi-center study it was shown that 33% of complications were missed in a time interval of 8 hours [26]. This could be an argument for a longer observation time period of 24 hours. However, only 1.7% of patients had a complication that needed hospitalization, but no further relevant treatment. Therefore, it was concluded that outpatient biopsies were not associated with an increased complication rate, which was later confirmed by other authors [27]. Even a reduction of the observation period to only 2 hours did not lead to an increased complication rate [28]. Therefore, kidney biopsies in an outpatient’s setting are now standard in most of the medical centers.

Early sonographic detection of compressing subcapsular renal hematomas is of vital importance for the prognosis [29]. Sensitivity of an ultrasound examination one hour after biopsy for detecting relevant complications was specified with 77% [30]. A renal hematoma of over 2 cm width that occurs directly after biopsy is a significant predictive value of a relevant bleeding [28]. In our patients relevant complications that needed immediate treatment, all appeared during the first 4 hours. All of them were detected with ultrasound or because of severe clinical symptoms which caused sonographic control.

Late complication of a venous oozing bleeding with relevant drop of hemoglobin that showed symptoms 3 weeks after biopsy had no further consequences except from blood transfusion. Two asymptomatic AV fistulas were detected in later ultrasound follow-up examinations, the time interval at which follow-up ultrasound examinations were performed was chosen depending on patient’s condition, symptoms and prompt post biopsy problems. Indication for interventional treatment necessities were always based on the development of ultrasound findings.

Even after changing from an inpatient to an outpatient setting no relevant complications between 4 and 24 hour post biopsy were observed. Hence, we recommend an early ultrasound follow-up examination 4 hours after biopsy. But there is a strong need for further follow-up to detect oozing bleedings and asymptomatic AV fistulas that might develop hemodynamic effects.

In conclusion percutaneous ultrasound-guided renal biopsy is an established and effective method in children and adolescents, but has a certain rate of complications and therefore should not be done without careful consideration.

Ultrasound examination is able to detect relevant complications and is essential for indication of interventional treatment (assessment of size and dynamics of a hematoma or a urinary obstruction). Especially in detection of asymptomatic AV fistula that need interventional treatment, ultrasound examination including Doppler is of great importance. However, no published standard operating procedure (SOP) is available. Therefore, development of such an SOP to avoid or at least to directly detect severe complications is definitively necessary.

We recommend ultrasound examinations (including Doppler) pre-, during, 4 hours post kidney biopsy and, in case no direct complication is visible, a few weeks after biopsy for preparation, execution and follow-up of kidney biopsies in children and adolescents. A first sonographic follow-up examination 4 hours post biopsy is suitable for outpatients and has a good predictive value for early occurring relevant complications. Especially patients with kidney transplants are at risk for developing AV fistulas, therefore Doppler examinations are important. As AV fistula could occur a few weeks after biopsy a long-term ultrasound examination including Doppler ultrasound is essential. Following such a center specific SOP we were able to achieve a low complication rate of 4.1%.
